# Depression and Sleep Quality among Iranian Women with Breast Cancer

**DOI:** 10.31557/APJCP.2021.22.11.3433

**Published:** 2021-11

**Authors:** Seyed Afshin Shorofi, Fereshteh Nozari-Mirarkolaei, Paul Arbon, Masoumeh Bagheri-Nesamie

**Affiliations:** 1 *Traditional and Complementary Medicine Research Center, Addiction Institute, Mazandaran University of Medical Sciences, Sari, Iran. *; 2 *Adjunct Research Fellow, Flinders University, Adelaide, Australia. *; 3 *Student Research Committee, School of Nursing and Midwifery, Mazandaran University of Medical Sciences, Sari, Iran. *; 4 *Torrens Resilience Institute, Flinders University, Adelaide, Australia; School of Nursing and Health Sciences, Flinders University, Adelaide, Australia. *

**Keywords:** Breast cancer, depression, sleep quality, BDI, PSQI

## Abstract

**Background and purpose:**

Breast cancer causes many psychological disorders such as sleep disturbances and depression. The current study was, therefore, intended to describe sleep quality and depression and to identify the association between these two psychological disorders among Iranian women with breast cancer.

**Materials and methods:**

This descriptive, analytical, cross-sectional study was carried out on 120 women with non-metastatic unilateral breast cancer undergoing chemotherapy in an outpatient chemotherapy unit of a major public hospital. A total of 120 women who had already undergone mastectomy procedure were selected via convenience sampling method. Data were collected by the Beck Depression Inventory-II (BDI-II) and the Pittsburgh Sleep Quality Index (PSQI).

**Results:**

The mean score on BDI-II was 13.40 (± 6.51), and 30% (n=36) of women had mild depression and 14.2% (n=17) reported moderate-to-severe depression. The mean global score of sleep quality was found to be 6.48 (± 2.62). Furthermore, 50.8% (n=61) of women obtained a global PSQI score of 5. A positive correlation was found between depression scores and sleep quality scores (p=0.001, r= 0.48). Depression was also correlated with age, number of children, household gross income, sleep duration, sleep latency, and type of mastectomy procedure (p<0.05). Moreover, subjective sleep quality was correlated with number of chemotherapy sessions (p=0.001, r=-0.67) and daytime dysfunction (p=0.001, r=0.78). A positive correlation was also observed between sleep disturbances and habitual sleep efficiency (p = 0.02, r = 0.65).

**Conclusion:**

In conclusion, 30% of women had mild depression and 14.2% reported moderate-to-severe depression. The mean global score of sleep quality was found to be 6.48 (± 2.62), suggesting poor sleep quality. Furthermore, over half of the participants (50.8%) obtained a global PSQI score of 5 or greater which is indicative of poor sleep quality. A positive moderate correlation was also observed between depression and poor sleep quality.

## Introduction

Breast cancer is the most common type of cancer in women with the highest mortality rate at the age of 40-44 years (Bray et al., 2018). There is an estimated 2.1 million new cases of breast cancer each year. In 2018, about 627000 women died from this cause worldwide, accounting for almost 15% of all cancer deaths among women (WHO, 2019). The fifth most common cause of cancer death, breast cancer is also the most frequent cancer in Iranian women, estimated to comprise 24.4% of all cancers. About 6160 new cases of breast cancer are diagnosed in women each year in Iran (Farhood et al., 2018). 

Breast cancer causes many problems for the patient. Psychological disorders such as sleep disturbances (Lowery-Allison et al., 2018), depression, anxiety (Park et al., 2018), helplessness, social withdrawal and stress (Bener et al., 2017) are some of these problems. Depression is one of the most common comorbidities of cancer patients (Mausbach et al., 2018). It seems that several factors are associated with depression in women with breast cancer, including age, educational attainment, lymphedema, self-esteem levels, body image (Boing et al., 2019), type of breast surgery (Boing et al., 2019; Rady et al., 2018), employment status, household income, obesity, marital status, side effects of chemotherapy on fertility, sexual function and perimenopausal period (Huang et al., 2019). Moreover, there is a significant association between depression and sleep disorder (Mansano-Schlosser et al., 2017). 

One of the most common complaints in patients with cancer is reported to be sleep disorders observed in about 50% of these patients, and most frequently in women and patients with breast cancer (Izci et al., 2016). Patients diagnosed with breast cancer report higher rates of sleep disorders (67% to 90%) compared with other cancer patients. In fact, these patients are two times more likely to report sleep problems than women free from cancer, and these problems may continue up to 10 years post-treatment. Sleep problems may lead to substantial adverse consequences, including “poor health-related quality of life, fatigue, poor healing, cognitive dysfunction, lost work productivity, safety issues (e.g., accidents), poor relationships, and increased health care costs” (Otte et al., 2016). 

Sleep disorders and depression commonly occur together in breast cancer survivors and interact with each other to negatively affect patients’ quality of life (Fiorentino et al., 2011). Sleep disorders, such as hypersomnia, short sleep duration, restless legs syndrome, parasomnia, sleep apnea and insomnia, experienced by breast cancer survivors were detected to be associated with decreased physical function and emotional states (including, depressive symptoms and distress), while low sleep quality was observed to be associated with greater levels of anxiety and depression (Cho and Hwang, 2021). However, “sparse population-based data exist about the contribution of insomnia symptoms to occurrence of depressive symptoms in breast cancer survivors” (Haque et al., 2020). A study by Ho et al., (2015) found a correlation between depression, fatigue and sleep quality in both premenopausal and postmenopausal women with breast cancer. Difficulties in falling asleep and waking up in the middle of the night have been found to be significantly associated with greater depression in breast cancer patients (Mansano-Schlosser et el., 2017). Depressive symptoms are also the identified predictors of subjective sleep disturbance in women diagnosed with breast cancer before, during and after radiation therapy (Dhruva et al., 2012). According to Ancoli-Israel et al., (2014), breast cancer patients had more severe symptoms than those without cancer even before undergoing chemotherapy. Women with breast cancer had significantly worse fatigue, more disrupted circadian activity rhythms, worse sleep and more depression symptoms at the end of four cycles of chemotherapy compared with their own baseline levels and cancer-free controls. Although the symptoms returned to baseline levels by one year, they were still worse than those in cancer-free controls. 

It is important to point out that both depression and sleep disorders are significantly associated with an increase of mortality rate (Bach et al., 2020). An association has been found between mild sleep disturbance and increased mortality in premenopausal women with breast cancer (Vaughn et al., 2018). Depression can also bring about a lower quality of life and seriously compromise patient outcomes, resulting in higher rates of mortality in cancer (Mansano-Schlosser et al., 2017). A systematic review and meta-analysis presented reasonable evidence that psychological distress (symptoms of depression and anxiety) is related to an increased mortality rate and poorer survival in patients with cancer (Wang et al., 2020).

According to the available databases, no study has been conducted in Iran to date on depression and sleep quality in women with breast cancer undergoing chemotherapy. The cultural differences in manifestations of depression (Falicov, 2018), different strategies used to cope with breast cancer (Sajadian et al., 2017; Kvillemo and Bränström, 2014), and the relevance of sleep-related behaviours to social and cultural factors (Grandner et al., 2013) may affect the outcomes of various studies conducted for the assessment of depression and sleep quality in patients with cancer. The current study was, therefore, intended to describe sleep quality and depression and to identify the association between these two psychological disorders among Iranian women with breast cancer.

## Materials and Methods

*Sample and sampling method*

This descriptive, analytical, cross-sectional study was conducted from December 2018 to September 2019. The study population consisted of women with breast cancer undergoing chemotherapy in an outpatient chemotherapy unit of a major public hospital affiliated to Mazandaran University of Medical Sciences (Sari, Iran). Eligibility criteria included women diagnosed with non-metastatic unilateral breast cancer aged ≥18 who had undergone mastectomy (any type) and were being treated with chemotherapy with no experience of stressful events (other than breast removal operation; e.g. death of a beloved one) within the last six months (Zwielewski and Sant’Ana, 2019), no history of depression or other mental disorders, and no history of taking sleeping medication and tranquillizers prior to the diagnosis of breast cancer. We needed a sample size of 120, which was based on a previous study (Musarezaie et al., 2014) and power analysis calculation. Participants were selected via convenience sampling method due to the relatively small population of patients with breast cancer. 

*Measurement instruments*

Data were collected using a socio-demographic/disease-related information form, the Pittsburgh Sleep Quality Index (PSQI), and the Beck Depression Inventory-II (BDI-II). The socio-demographic/disease-related information form collected data on age; number of children; native or non-native to Mazandaran province; marital status; educational attainment; occupation; household gross income; emotional support resources; side of involved breast; type of mastectomy procedure; time elapsed since mastectomy; number of chemotherapy sessions received; history of depression, sleep disturbance, hospital admission; family history of breast and other cancers; and history of taking sleeping medication. 

A commonly used indicator of the severity of depression, the BDI-II is a self-report inventory consisting of 21 sets of statements, each ranked on the basis of severity and scored on a scale value of 0 to 3. The total score ranges from 0 to 63, with higher scores indicating greater severity of depression. Scores of 0-13 indicate minimal depression, 14-19 mild depression, 20-28 moderate depression, and 29-63 severe depression (Smarr and Smarr, 2011). The Persian version of BDI-II has demonstrated acceptable psychometric properties of validity and reliability in different populations (Hamidi et al., 2015; Ghassemzadeh et al., 2005; Ahmadi et al., 2019; Rahimi, 2014).

The PSQI, developed by Buysse et al., (1989), is a valid and reliable instrument for the assessment of sleep quality and disturbances in the previous month. The PSQI has demonstrated a high degree of internal consistency with Cronbach’s [alpha] coefficient of 0.83 and validity of 0.75. In addition, the Persian version of PSQI has also been reported to have optimal validity and reliability in different populations (Farrahi Moghaddam et al., 2012; Mohammad Gholi Mezerji et al., 2017; Nazifi et al., 2014). The PSQI consists of 19 self-rated questions which evaluates 7 sleep components including subjective sleep quality, sleep latency, sleep duration, habitual sleep efficiency, sleep disturbances, use of sleeping medication, and daytime dysfunction. Each component is weighted equally on a 0-3 scale, with a global score ranging from 0 to 21. A global score of 5 or greater is indicative of poor sleep quality (Buysse et al., 1989).

*Procedures*

 Participants were selected by convenience sampling method and recruited to the study. Socio-demographic/disease-related characteristics were collected from participants through face-to-face interviews and patients’ medical files. Depression was measured using the BDI-II and sleep quality was evaluated with the PSQI. All data collection tools were completed in the presence of the researcher.

*Data analysis*

The collected data were coded and statistically analyzed with SPSS 22.0 software (SPSS, Inc., Chicago, IL) using descriptive statistics (mean, percentage and standard deviation) and analytical tests (Chi-square and Pearson’s correlation coefficient). The significance level was set at a p value of <0.05.

*Ethical considerations*

The study protocol was approved by the Bioethics Committee of Mazandaran University of Medical Sciences (MAZUMS.IR.S.261) in accordance with the Helsinki Declaration in its latest version. Additional access approvals were obtained from the participating hospital. Our participants were initially properly informed about study aims, procedures, and confidentiality. They were also assured that they would be free to withdraw from the study at any time without giving a reason and without any adverse effect on their continued care. All participants signed an informed consent form in which the study procedures and their rights had been delineated.

## Results

*Sociodemographic and disease-related characteristics*

A total of 120 patients who met the selection criteria participated in the study with a mean age of 45.7 (±4.52) years (range: 29-63 years). Less than half of the participants (n = 56, 46.7%) stated that they were in their menopausal period. In addition, 45.8% of women had a family history of cancer. The average time elapsed since mastectomy was 5.52 (±3.5) months. Patients’ socio-demographic/disease-related characteristics are summarized in Table 1. 

*Levels of sleep and depression*

The mean score on BDI-II was 13.40 (± 6.51). Approximately one third of women (n=36, 30%) had mild depression and 14.2% (n=17) reported moderate-to-severe depression (Table 2). The mean global score of sleep quality was also found to be 6.48 (± 2.62). Furthermore, over half of the participants (50.8%, n=61) obtained a global PSQI score of 5 or greater which is indicative of poor sleep quality. Table 3 depicts the mean (± standard deviation) scores on global PSQI and PSQI subscales.

Negative correlations were also found between depression and age (p= 0.002, r = - 0.72), number of children (p = 0.02, r = - 0.54), and household gross income (p = 0.003, r = - 0.65). As such, younger patients, those with fewer children and patients with lower household gross income were more likely to experience higher levels of depression. In addition, the Chi-square test indicated that there was a significant relationship between depression and type of mastectomy procedure (p= 0.04) so that women with total and radical mastectomy were more likely to report higher levels of depression than the others. The Pearson’s correlation coefficient showed that there was a significant negative correlation between number of chemotherapy sessions and subjective sleep quality in breast cancer patients (p=0.001, r=-0.67). Moreover, subjective sleep quality was positively significantly correlated with daytime dysfunction (p = 0.001, r = 0.78). A positive correlation was also observed between sleep disturbances and habitual sleep efficiency (p = 0.02, r = 0.65).

*Association between sleep quality and depression*

**Figure 1:**
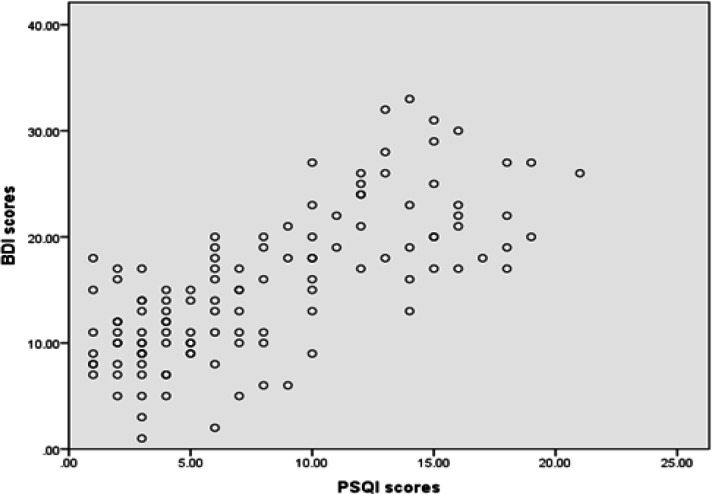
The Scatter Graph Showing a Positive Moderate Correlation between PSQI Scores and BDI Scores. BDI scores, Beck Depression Inventory scores; PSQI scores, Pittsburgh Sleep Quality Index scores

The Pearson’s correlation coefficient showed a positive moderate correlation between depression scores and sleep quality scores (p = 0.001, r = 0.48) (Figure 1). Furthermore, depression was positively correlated with sleep duration (p = 0.01, r = 0.58) and sleep latency (p = 0.02, r = 0.49). 

**Table 1:** Socio-Demographic/ Disease-Related Characteristics of the Participants (n=120)

Characteristics		Characteristics	
Age	(Years, M ± SD)	Menopausal symptoms	n (%)
	45.7 (±4.52)	Yes	56 (46.7)
		No	64 (53.3)
Native of Mazandaran province	n (%)		
Yes	114 (95)	Emotional support resources	n (%)
No	6 (5)	Spouse	52 (43.3)
		Parents	24 (20)
Number of children	n (%)	Children	33 (27.5)
0	4 (3.3)	Other	11 (9.1)
1	11 (9.2)	Family history of cancer	n (%)
2	47 (39.2)	Yes	55 (45.8)
3	45 (37.5)	No	65 (54.2)
4	4 (8.3)		
5	2 (1.7)	Family history of breast cancer	n (%)
6	1 (0.8)	Yes	48 (23.3)
		No	92 (76.7)
Marital status	n (%)		
Single	2 (1.7)	History of hospital admission	n (%)
Married	109 (90.8)	Yes	46 (38.3)
Divorced/separated	3 (2.5)	No	74 (61.7)
Widow	6 (5)		
		Involved breast	n (%)
Educational attainment	n (%)	Right	62 (51.7)
Academic degree	33 (27.5)	Left	58 (48.3)
Diploma and lower	87 (72.5)		
		Type of mastectomy procedure	n (%)
Employment	n (%)	Partial	75 (62.5)
Housewife	96 (80)	Radical	9 (7.5)
Employee	6 (5)	Total	36 (30)
Self-employed	15 (12.5)		
Student	1 (0.8)	Time elapsed since mastectomy	(years, M ± SD)
Unemployed	1 (0.8)		5.52 (±3.5)
Retired	1 (0.8)	Number of chemotherapy sessions	n (%)
		<5	66 (55)
Household gross income	n (%)	>5	54 (45)
Good	6 (5)		
Moderate	22 (18.3)		
Low	92 (76.7)		

M ± SD: mean ± standard deviation; n (%): number (percent).

**Table 2:** Depression Scores in Women with Breast Cancer

BDI scores	Level of depression	n (%)	Range	Mean(±standard deviation)
0-13	Minimal depression	67(55.8)	2-12	
14-19	Mild depression	36(30)	14-19	13.40 (± 6.51)
20-28	Moderate depression	15(12.5)	20-26	
29-63	Severe depression	2(1.7)	29-38	

BDI-score, Beck Depression Inventory-score

**Table 3:** Mean (± standard deviation) Scores on Global PSQI and PSQI Subscales

Subscales	Mean (± standard deviation)	Range
Subjective sleep quality	1.34 (± 0.9)	0-2
Sleep latency	1.07 (± 0.3)	0-3
Sleep duration	0.74 (± 0.4)	1-3
Habitual sleep efficiency	2.16 (± 1.5)	1-3
Sleep disturbances	1.47 (± 0.6)	0-3
Use of sleeping medication	0.40 (± 0.2)	1-2
Daytime dysfunction	0.85 (± 0.7)	0-2
Global PSQI	6.48 (± 2.62)	2-15

PSQI, Pittsburgh Sleep Quality Index

## Discussion

This study examined the relationship between depression and sleep quality in Iranian women with breast cancer. Nearly half the participants (44.2%) reported having mild-to-severe depression, which is considerably higher than the prevalence of depression among Iranian women reported by Musarezaie et al., (2015) and Noorbala et al., (2017). The prevalence of depression among women with breast cancer was reported in 2017 to be 44% in the USA (Park et al., 2018). It is reasonable to assume that differences in socioeconomic status including age, educational attainment (Boing et al., 2019; Freeman et al., 2016), income level (Huang et al., 2019; Freeman et al., 2016) and marital status (Rady et al., 2018; Huang et al. 2019, Freeman et al., 2016); religiosity and religious coping (Ng et al., 2017); coping patterns (Li et al., 2017); menopausal state; family history of breast cancer; breast cancer staging; type of breast surgery (Boing et al., 2019; Rady et al., 2018); diagnosis of other diseases; lymphedema; self-esteem; body image (Boing et al., 2019); obesity; and side effects of chemotherapy on fertility and sexual function (Huang et al., 2019) may explain inconsistencies in the reported prevalence of depression among women with breast cancer by different studies. Depression may itself bring about many negative consequences, including longer hospital stays, increased physical distress, poorer adherence to treatment, lower quality of life, and high desire for hastened death (Li et al., 2016). Therefore, various and effective strategies (such as psychosocial interventions) are required to prevent and treat co-morbid depression and poor mental health among women with breast cancer. Psycho-oncologists can play a critical role in the screening, assessment and management of depression in this group of patients. They may use combined approaches (including psychotherapy and psychopharmacology) to manage, prevent and reduce the distress and psychosocial morbidity associated with cancer. Other healthcare professionals, particularly oncologists, nurses and social workers, can also provide patients and their families with psychological, social and emotional support, and help them cope with the demands of treatment and the uncertainty over the course of treatment and prognosis, and improve their quality of life.

Our results also demonstrated a relationship between depression and type of mastectomy procedure so that women with total and radical mastectomy were more likely to report higher levels of depression than their counterparts with partial mastectomy. Boing et al. (2019), for example, concluded that breast surgery, specifically radical mastectomy, was associated with depression symptoms. A systematic review and meta-analysis also found no significant differences among three types of surgery (total mastectomy, breast conserving therapy, and breast reconstruction) on depression in female breast cancer patients (Zhang et al., 2018). Notwithstanding a relationship between type of mastectomy procedure and depression in our study, it is still difficult to conclusively determine that our participants’ depression was caused by cancer or mastectomy (Kim et al., 2017).

 Younger patients, those with fewer children and patients with lower income in our study were more likely to experience higher levels of depression. It is possible that younger breast cancer women experience more disruption in body image, anxiety, sleep disorders, dissatisfaction with relationships, fear of relapse (Mendoza et al., 2017), worse sexual problems (Mendoza et al., 2017; Jankowska, 2013), higher rates of depression, poorer quality of life and psychological well-being than older counterparts (Jankowska, 2013). They may also have relatively higher concerns about loss of fertility and premature menopause (Jankowska, 2013). It is possible that women with fewer children in the present study were receiving less support than women with more children, which could have a negative impact on their mental health. Children could be an important source of support for these highly stressed women. Christie et al., (2010) found that single breast cancer women, who were considered to feel lonely and deprived of emotional support, reported higher depressive symptoms. Our finding that women with lower income were more likely to have higher depression than those with higher income is in line with the extant literature (Chen et al., 2009; Srivastava et al., 2016; Wei et al., 2019).

The findings of the present study indicate that over half of the women with breast cancer (50.8%) had poor sleep quality. The prevalence of poor sleep quality in breast cancer patients varies greatly, depending on study design, sleep disturbance scale used, different clinical and demographic profiles of samples studied, and type of cancer treatment received by women (Khorami et al., 2012; Berger et al., 2019; McManus et al., 2019; Alifiyanti et al., 2018; de Araújo et al., 2014; Carroll et al., 2019; Vin-Raviv et al., 2018; Gonzalez and Lu, 2018). We also found a positive moderate correlation between depression and poor sleep quality. There is a complex relationship between sleep disturbance and mood state because sleep disturbance can result in such psychological disorders as depression, anxiety and emotional changes, and these conditions can in turn interfere with sleep (SleepFoundation.org, updated 18 September 2020). Sleep disturbances and depression which can co-occur as part of symptom clusters in these patients (Ho et al., 2015) can be lasting and have a negative impact on patients’ quality of life. Therefore, effective management of sleep disturbances and psychological disorders in women with breast cancer is of great importance.

This study has several limitations. The current study was conducted on post-mastectomy women with non-metastatic unilateral breast cancer undergoing chemotherapy in an outpatient chemotherapy unit of a major public hospital affiliated to Mazandaran University of Medical Sciences (Sari, Iran), which may limit the generalizability of the findings. Another potential limitation of this study pertains to a relatively small sample size of patients, although it is important to consider that the adequacy of the sample size was ensured by power analysis calculation. Studies with larger sample sizes would certainly impart more power to analyze the various variables reported. Another potential limitation of the present study is that the findings are based on data from Iranian women with low socioeconomic status which may limit the generalizability of the data. The final limitation is the use of self-report measures which may have not reflected participants’ actual sleep quality and depression.

Several insights were obtained from this study. Effective management of sleep disturbances and depression should be one of our clinical priorities for women with breast cancer. More attention should also be paid to depression and sleep disturbances by healthcare professionals, particularly nurses and physicians, providing care and medical treatment for women with breast cancer. In addition, oncology social workers have an important role in helping these patients with practical needs, like finding sources and financial assistance. It is also worth noting that coping strategies adopted by Iranian cancer patients mainly stem from their religious beliefs that should be respected by healthcare professionals as spiritual and religious beliefs have protective effects against depression (Mahdanian, 2018).

In conclusion, 30% of women had mild depression and 14.2% reported moderate-to-severe depression. The mean global score of sleep quality was found to be 6.48 (± 2.62), suggesting poor sleep quality. Furthermore, over half of the participants (50.8%), obtained a global PSQI score of 5 or greater which is indicative of poor sleep quality. In addition, a positive moderate correlation was observed between depression and poor sleep quality. Depression and sleep quality were also associated with diverse socio-demographic factors. One area for future research is to explore the relationship between coping with cancer diagnosis, depression and sleep quality among women with breast cancer. Future research should also incorporate both subjective and objective measures of sleep (such as polysomnography and actigraphy plus physician/nurse- or patient-administered tools) and depression (such as laboratory methods plus physician/nurse- or patient-administered tools) in breast cancer survivors.

## Author Contribution Statement

SA. Shorofi: Conceptualization; Data Curation (supporting); Funding Acquisition (equal); Investigation (equal); Methodology; Project Administration; Supervision; Validation; Visualization; Writing – Original Draft (lead); Writing – Review & Editing. F. Nozari: Formal Analysis (equal), Funding Acquisition (equal), Investigation (equal), Writing – Original Draft (supporting), Data Curation (lead). P. Arbon: Writing – Original Draft. M. Bagheri-Nesami: Formal Analysis (equal).

## Funding statement

This study was funded by Mazandaran University of Medical Sciences, Sari, Iran. 

## Conflict of interest

The authors declare no conflict of interest.
